# The Rapid interaction: a qualitative study of provider approaches to implementing Rapid ART

**DOI:** 10.1186/s43058-023-00464-w

**Published:** 2023-07-14

**Authors:** Lissa Moran, Kimberly A. Koester, Noelle Le Tourneau, Susa Coffey, Kelvin Moore, Janessa Broussard, Pierre-Cedric Crouch, Lyndon VanderZanden, John Schneider, Elizabeth Lynch, Jorge Roman, Katerina A. Christopoulos

**Affiliations:** 1 Department of Medicine, University of California, 550 16Th Street, San Francisco, CA USA; 2 Department of Medicine, Zuckerberg San Francisco General Hospital, University of California, San Francisco, San Francisco, CA USA; 3 Department of Community Health Systems, School of Nursing, University of California, 2 Koret Way, San Francisco, CA USA; 4 Howard Brown Health Center, 4025 N. Sheridan Rd, Chicago, IL USA; 5 Department of Medicine, University of Chicago, 5841 South Maryland Street, Chicago, IL USA; 6 San Francisco AIDS Foundation, 470 Castro Street, San Francisco, CA USA

**Keywords:** Rapid ART, Same-day start, Patient-provider interaction, Provider messaging, Provider narratives, Individual-level implementation, CFIR

## Abstract

**Background:**

Offering antiretroviral therapy (ART) to patients directly following an HIV diagnosis (“Rapid ART”) improves clinical outcomes and is feasible and acceptable for patients and providers. Despite this, implementation of Rapid ART is not yet standard practice in the USA. Structural-level implementation guidance is available, but research at the individual provider level that explores the patient-provider interaction itself remains scarce. The Consolidated Framework for Implementation Research (CFIR) provides a nuanced guide to investigating the less visible, more social elements of implementation like the knowledge and feelings of people, and the influences of culture and resources on individual approaches.

**Methods:**

We conducted a multi-site qualitative study, exploring intervention commonalities across three HIV clinic environments: an HIV primary care clinic; an HIV/STI testing, treatment, and prevention clinic; and a large federally qualified health center (FQHC). Qualitative data were gathered from 27 provider informants—Rapid ART program staff and clinicians—using an interview guide developed using the CFIR. An experienced qualitative team conducted a comprehensive thematic analysis and identified cross-cutting themes in how providers approach and engage in the Rapid interaction, as well as longer-form narratives from providers that describe more fully what this interaction looks like for them.

**Results:**

Three main themes represent the range and content of individual provider approaches to the Rapid interaction: (1) patient-centeredness; (2) emotional support and partnership; and (3) correcting misperceptions about HIV. Each theme encompassed both conceptual approaches to offering Rapid ART and concrete examples of messaging to the patient that providers used in the Rapid interaction. We describe and show examples of these themes, offer key take-aways for implementation, and provide expanded narratives of providers’ personal approaches to the Rapid interaction.

**Conclusions:**

Exploration of provider-level approaches to Rapid ART implementation, as carried out in the patient-provider Rapid interaction, contributes a critical layer of evidence for wider implementation. It is our hope that, together with existing research showing positive outcomes and core components of systems-level implementation, these findings add to an instructive body of findings that facilitates the implementation of Rapid ART as an enhanced model of HIV care.

**Supplementary Information:**

The online version contains supplementary material available at 10.1186/s43058-023-00464-w.

Contributions to the literature

- Despite robust evidence supporting ART initiation at the time of HIV diagnosis, “Rapid ART” has not been broadly adopted as a standard of care in the USA.
- Much of Rapid ART implementation takes place within the patient-provider interaction, but there is a gap in individual-level implementation research; the content of these ‘Rapid interactions’ is not well understood.
- This qualitative analysis of provider approaches to the Rapid interaction uses the CFIR to explore social and interpersonal elements of implementation.
- Findings are instructive at the individual staff and provider level and contribute a critical layer of evidence for wider Rapid ART implementation.

## Background

### Rapid ART

Initiation of ART on, or as close as possible to, the day of HIV diagnosis has been shown to improve clinical outcomes, to be acceptable for patients and providers, and to be feasible for healthcare systems [1–5]. While the precise definition of what constitutes rapid start ART (henceforth “Rapid ART”) varies across studies and settings [6], evidence going back more than a decade suggests that from clinical, behavioral, and programmatic perspectives, the closer ART initiation is to HIV diagnosis, the better [1–3, 7–18]. For the purposes of this study, we borrow an early and inclusive definition of Rapid ART: “Rapid antiretroviral therapy (ART) means initiating ART as soon as possible after diagnosis of HIV, ideally on the day of diagnosis and, if not then, on the day of entry to care (studies of rapid ART often use a metric of ≤ 7 days from diagnosis)” [19].

A growing body of evidence shows Rapid ART to be safe [8], effective [8, 9, 20], and a potential facilitator to epidemic control [10]. Early ART initiation protects patients diagnosed during early acute HIV infection from irreparable harm to their immune system [11, 21, 22], can play an important role in transmission prevention [2, 12, 23], and can be life-saving for patients with untreated advanced disease [1, 13]. As an intervention, Rapid ART has been shown to improve patient ART uptake, retention in care, and time to viral suppression compared with standard ART initiation [14–16, 19, 20]. Globally, early ART initiation is recommended as a standard of care for new HIV diagnoses [24–26], and a 2022 meta-analysis of 10 Rapid ART studies further supports broad application of the model [17].

Despite this robust evidence base, adoption of Rapid ART programming was initially slow to gain traction in the USA and remains a topic of debate among some providers. Public forum debates have centered on a lack of consensus on how to interpret the evidence base (e.g., whether global studies on Rapid ART outcomes can or should be applied to the USA; which outcome metrics are more reflective of program success—retention in care or viral suppression), whether the relative merits of decreasing time from HIV diagnosis to treatment initiation outweigh the risks presented by initiating ART prior to lab screenings, and differing perspectives on whether early ART initiation decreases or increases the risk of patient loss to follow-up [27].

### Implementation and the “Rapid interaction”

Structural issues can complicate Rapid ART implementation; access to ART and elements of linkage to care can differ state to state [28] and organizational readiness for implementation relies on multiple elements [29, 30]. At the individual level, however, provider hesitancy may be rooted in a knowledge gap between the known of traditional ART initiation, and the unknown of what the Rapid ART model looks like in practice. Indeed, much of Rapid ART implementation occurs behind closed doors, often between two people, one of whom has just been given an HIV diagnosis and the other of whom has been entrusted with their care. We are calling this dynamic the “Rapid interaction.”

Behavioral HIV research highlights the importance of the patient-provider interaction. A 2015 dyadic study identified the patient-provider encounter as “a key factor in ART usage” [31],while other studies of HIV provider messaging and patient experience suggest that even a single conversation can make a big difference in the choices that patients make about their treatment [32]. Researchers studying Rapid ART specifically have stressed “a need for delivery of the message to start treatment tailored to the individual patient” [28]. That said, it is not well understood how staff and providers can cultivate this trust and connectedness within the unique context of the Rapid interaction.

Few US studies explore uptake of Rapid ART, and those that do focus on patient medication uptake, not how or why providers or clinics choose to implement the Rapid ART model. Prior research has called for further investigation, not just of Rapid ART outcomes, but of *how* these programs are delivered [33].

### Study and aims

This study examined how the providers and staff who work directly with patients operationalize and carry out Rapid ART implementation. We explored implementation commonalities across three different HIV clinic environments: an HIV primary care clinic in a public health setting; an HIV/STI testing and prevention site; and a large federally qualified health center (FQHC).

System-level challenges and facilitators to implementing Rapid ART were published in 2022 [29], while this analysis focuses on implementation at the individual staff and provider level. The purpose of this analysis was to better understand how staff and providers carried out Rapid ART: how they approach the idea conceptually; how they present the offer of same-day ART initiation to patients; and how they support, inform, and equip patients to make informed decisions about how and when to initiate treatment. Specific aims were to (1) identify cross-cutting thematic commonalities in how staff and providers approach and engage in the Rapid interaction, and (2) present longer-form first-person narratives from staff and providers that describe the layered fullness of how this interaction takes shape in their practice.

### The consolidated framework for implementation research

At its best, the Consolidated Framework for Implementation Research (CFIR) can provide a bridge between observable structural features of implementation and less visible, more social elements—like the knowledge and feelings of people, and the influences of culture, resources, and organizational dynamics on individual approaches. It seeks to shine a light into the corners of implementation realities that are difficult to capture, but which can account for the distance between implementation intention and practice.

The CFIR invites one to explore a program or intervention through the lens of 37 unique and dynamic constructs, organized across five domains: intervention characteristics; the outer setting; the inner setting; characteristics of the individual; and implementation process. The CFIR further distinguishes between program elements that are “core components”—essential elements that comprise the DNA of a program—versus the more variable tailoring elements along the “adaptable periphery.” It is here—the adaptable periphery—that we frame our analysis, aiming to elucidate the common themes that arise across the inherently variable individual deployments of a model that is ultimately defined more by a single output (same-day or accelerated initiation of ART) than by a uniform process to achieve it. We assert that this framing honors the original intention of the CFIR as not just a consolidated framework, but one that contributes to the forward movement of the implementation science field.

Laura Damschroder’s seminal paper introducing the CFIR [34] was concerned both with large structural and organizational influences on implementation, as well as with what she called the “dynamic interplay between individuals,” and the ways that the granular realities of implementation processes, as performed by people, may be “formally planned or spontaneous; conscious or sub-conscious; linear or non-linear.” She quotes Greenhalgh’s reflection on the influence of individuals implementing innovations from the review that served as the foundation of the CFIR’s development [35]:

People are not passive recipients of innovations. Rather....they seek innovations, experiment with them, evaluate them, find (or fail to find) meaning in them, develop feelings (positive or negative) about them, challenge them, worry about them, complain about them, “work around” them, gain experience with them, modify them to fit particular tasks, and try to improve or redesign them – often through dialogue with other users.

When critical elements of an implementation happen not in collaboration with “other users,” but instead within the intimacy of a sensitive patient-provider encounter, diffusion of skills and best practices can be constrained, particularly when the total population of implementing providers is relatively small. In such cases, as in the case of Rapid ART, potential implementers may rely on guidance from existing research and literature. Contributing rare and valuable first-person insights of experienced Rapid ART staff and providers to the literature was a key motivation of this analysis.

## Methods

### Study design and setting

As part of a multi-site qualitative study, we conducted in-depth interviews with clinical providers and staff who held operational roles in executing Rapid ART in three clinical environments. The study was initially designed with a focus on the HIV primary care clinic in San Francisco that first formally developed the “RAPID” (Rapid ART Program for Individuals with an HIV Diagnosis) model [15], subsequently adding two sites that had adopted the model to operate in different clinical contexts. One site was an HIV and STI testing, treatment, and prevention clinic, also in San Francisco, and the other was a large FQHC in Chicago.

### Data collection

Qualitative data were collected from March 2018 to February 2020. Eligible participants included clinic staff, clinicians, or administrators with a direct role in the Rapid ART program at each site.

Sampling and participant recruitment took place in two stages. The first stage was purposive sampling, in which known Rapid ART implementers at each site were contacted and invited to participate in an in-depth interview, approximately one hour in length. From that point, we employed snowball sampling, where those initial key informants recommended additional potential informants to contact, and those informants recommended others, etc. Prospective participants were offered a small incentive ($20 gift card) in appreciation for their time. Only one prospective participant declined to be interviewed, citing a lack of schedule availability.

The interview guide was developed by KK and KAC, using selected CFIR constructs across all 5 domains as content guides. In addition to questions that investigated implementation elements at the structural and organizational levels, we focused several questions on how the informant personally approached their role in the Rapid interaction, querying their opinions and feelings, examples of “difficult” and “successful” interactions, and to “walk [us] through” how they discuss Rapid ART with a client (interview guide available upon request). Responses to these questions ultimately yielded implementation elements at the individual staff and provider level.

The qualitative team consisted of three analysts with deep and varied experience in both qualitative methods and implementation science (KAK, NL, LM). The analysts were not healthcare providers themselves, did not work for any of the participant organizations, nor were they people living with HIV.

In-depth interviews were conducted both in person and over the phone by KAK and LM, who each have over a decade of experience interviewing clinical staff and providers within the context of HIV prevention and care. Because the study team was based in San Francisco, nearly all (20 out of 22) local interviews were conducted in person, while interviews with participants in Chicago were conducted over the phone.

### Characteristics of participants

We interviewed 27 staff and providers across three service sites: 10 from the HIV Primary Care clinic, 12 from the HIV and STI testing, treatment, and prevention site, and 5 from the FQHC. Of these participants, 15 (56%) were women, 10 (37%) were men, and 2 (7%) self-defined as trans or non-binary. Of note, we invited participants to self-report their gender and did not require that they disclose if they were cisgender or transgender; we do not make assumptions about their sex assigned at birth. Participants were majority White; 19 participants (70%) were White, 6 (22%) were Latinx, 1 participant (4%) was Asian, and 1 participant (4%) was Black.

Participant roles were close to evenly split between clinicians and program staff. We interviewed 8 (29%) prescribing providers (MD, NP, or PharmD), 4 (15%) registered nurses, 11 (41%) program specialists (e.g., social workers, linkage specialists, navigators), and 4 (15%) individuals who served in both leadership and prescribing provider roles.

### Analysis

The qualitative team conducted a comprehensive thematic analysis. Following each interview, the interviewer composed a fieldnote capturing extemporaneous observations, impressions, and context, as well as a summary of the interview based on notes. All three analysts reviewed and summarized transcripts of interviews conducted with participants from the first site—the HIV primary care clinic where the RAPID model originated —and consolidated summaries into a comprehensive analytical memo which, along with the CFIR, served as the basis for preliminary code book development.

Transcripts of each interview with participants from the subsequent adopter sites—the testing and prevention site and the FQHC—were uploaded into the qualitative data management computer program Dedoose [36]. The qualitative team collectively reviewed a transcript selected for its data richness and further refined the code book as a team. Each analyst then independently reviewed the same 2 additional transcripts and met to cross-check coding choices, discuss areas of coding concordance and discordance, and further refined the code book (available upon request) by adding, dropping, or expanding upon codes. The remaining transcripts were divided among the team, each assigned a primary and secondary coder.

Following coding, the team reviewed code reports downloaded from Dedoose, and generated code summaries individually or as a team, depending on the complexity of the data captured in a single code.

This analysis was comprised of both deductive and inductive elements. We first used the CFIR deductively, where it guided the scope of the data collected as well as the preconceived domains and constructs that informed the interview guide and some a priori codes. Additional themes were generated inductively, encompassing both new themes (e.g., patient-provider interactions), as well as the content of data findings elicited from CFIR themes (e.g., adaptability, relative advantage). The analysis continued to cycle through inductive and deductive processes as we homed in on our findings.

We held a report back session (member check) with a subset of key informants—staff and providers from the first site—to share preliminary findings [37]. Members provided feedback on what of our findings they felt would be maximally useful to communicate to other implementing sites, and corrected some of our colloquial language that held clinical meaning (e.g., “triage”).

To capture provider narratives of the Rapid interaction, we reviewed excerpts that had been coded with any code linked to the *Characteristics of the Individual* and *Implementation Process* domains of the CFIR. We identified which codes effectively captured the data within our scope of interest, then narrowed down the codes to 4 to review and re-summarize where appropriate. We summarized excerpts first by site, noting and clustering themes within each sample. The next steps were iterative, as we compared themes across sites, consolidated excerpts by theme and sub-theme, then revised themes and re-organized excerpts, until generating the final classification of 3 care themes. To aide in the utility and digestibility of our findings, we extrapolated key take-aways for implementation—a brief synthesis of key approaches or strategies offered by participants.

See Additional file 1 for codes reviewed and excerpted and the progression of theme generation. The completed Standards for Reporting Qualitative Research (SRQR) checklist [38] is included as Additional file 2.

## Findings

Through this analysis, three main themes represent the range and content of approaches to the Rapid interaction as reported by informants: (1) patient-centeredness; (2) emotional support and partnership; and (3) correcting misperceptions about HIV. Each theme encompassed both *conceptual approaches* to offering Rapid ART, and concrete examples of *messaging to the patient* that participants reported consistently saying or doing in the Rapid interaction. To enhance the utility of these findings, we further offer a summary of key take-aways for implementation following each theme.

For this report, we are presenting two types of excerpts from the qualitative data: short representative excerpts illustrative of themes identified across sites; and longer-form excerpts that showcase the fullness of the Rapid interaction as described by participants. The former excerpts are included in the presentation of themes below. The latter excerpts contain multiple interwoven themes and are included in Additional file 3.

Due to the small size of teams and our high threshold for maintaining participant confidentiality, we are unable to attribute participant excerpts to specific roles or credentials. Instead, we attribute excerpts by site (FQHC, HIV Primary Care, Testing Site) and whether the participant was a clinical provider or staff member.

### Patient-centeredness

#### Conceptual approach

Common across participant narratives was a concerted, intentional centering of the patient in the clinical encounter—their experience, their needs, and their wishes. Participants described, some using specific examples and some speaking generally of their approach, how varied the needs of Rapid ART patients are in the moment, and how those needs and that moment guided their approach to the Rapid interaction.

I usually try to make space for the patient to talk pretty early. Because that guides kind of what I’m going to say. If their main concern is a partner or their mom’s reaction or something like that, then the conversation’s going to go differently than if their main concern is their own body and their own health. [FQHC_Provider_05]

Importantly, informants stressed that, while there may be topics that are important to cover and ways of talking about a new diagnosis that they tend to use, there is no one script or protocol for a Rapid interaction.

[The approach] definitely varies. It’s based on what the patient is experiencing, I feel like. Sometimes people ask us immediately. They come in and they’re like, I want meds. Get me meds now. And we’re like, okay, great. This is what we’re going to do. Some people are too overwhelmed to ask questions. [HIV Primary Care_Staff_05]

The following participant framed client-centeredness as a harm reduction tool, and spoke about how they allowed it to challenge the assumptions and biases that they as the provider might bring into the room:

The thing is, in health care there’s a certain point where we [can] get paternal, and you don’t want to provide paternal care. Having done Hep C care and treatment for people who are homeless, actively using substances, and have a whole lot of issues, they do very well, and people cure Hep C. And they’re people who I never in a million years would’ve thought would be successful at it, and they have been. So, a lot of it was checking my own personal biases that I have. My philosophy is, it’s client-centered. The client decides what they want to do. And to be quite honest, if like one out of ten folks ended up not really being ready, I’m like, we’ll get them into care eventually, or hopefully we’ll get them when they’re ready. But I don’t want to dismiss the nine out of ten people who ended up starting care. [Testing Site_Provider_04]

#### Messaging to the patient

In addition to describing their mindsets of centering the patient, informants provided examples of things they say to patients to establish the patient as the driver of the Rapid interaction and of the decisions that they will be making.

So, I would never tell someone they can or they can’t. I mostly say, hey, is this something that you want to do? You could start today. You can also wait and start a different time. So, it’s letting them decide what’s right for them. [Testing Site_Provider_04]

These messages may serve multiple purposes—primarily to establish the patient as the center of the interaction, but also to build trust, provide reassurance, and educate patients about their options so that they can make an informed choice.

There’s certain like radical statements you can make where patients start to trust you immediately, like telling someone, you’re the boss of the experience. We’re going to give you information, and you decide what you want to do. [HIV Primary Care_Staff_05]

I also do tell them that “if you don’t feel ready right now, if you would prefer to come back at a later date, I could help schedule you an appointment to see a provider and start at that point.” Generally speaking, it’s not like we’re necessarily trying to talk up same-day start. But we do try to steer them in that direction just because getting on a medication quickly can start getting them to undetectable quicker. So, just being honest with them about their options, but also being like, “And this is what we would recommend based upon a lot of the success stories we’ve already had in people who have gone through same-day start.” [FQHC_Provider_04]

##### Key take-aways for implementation

➣ Follow the patient’s lead. Each patient will be coming to the Rapid interaction with different needs.

➣ Timing of ART initiation is ultimately the patient’s choice: “Is this something you want to do?”.

➣ Centering patient choice is not in conflict with providing medical guidance; it is still the provider’s job to recommend a treatment plan supported by evidence. Sample language: *This is what we recommend based upon the most up-to-date medical research and a lot of the success stories we’ve had with people who have gone through same-day start.*

### Partnership and emotional support

#### Conceptual approach

In interviews, participants acknowledged that receiving an HIV diagnosis and (simultaneously) considering treatment options could be overwhelming for patients, and that support throughout that process—emotional and logistical—was a great benefit of Rapid ART that was not included in the same way with delayed ART initiation.

Every handoff should be a warm handoff. … I think part of my job is to provide that emotional support and also to make sure that the red carpet is rolled out for them and that there’s - basically, no time where they're sitting alone. [HIV Primary Care_Staff_09]

Sitting with the client to me is one of the most important things, and establishing some rapport so that they can become increasingly comfortable to work with me and share some information that helps me figure out what's an appropriate medical home for them. [Testing Site_Staff_03]

One provider pointed out how establishing partnership in the Rapid interaction helped patients mentally bridge the experience of taking medication from the clinic to the home environment.

I think [it] show[s] a patient that [treatment] isn’t a scary thing. When they’re in the room with you, swallowing these pills, and you’re looking calm at them, it’s showing them that there’s not anything frightening or horrible about taking them - if they weren’t to do that in the clinic, they might go home and just start looking at those tablets and start thinking too much about them and thinking, “I can’t really do this. I can’t really do this.” Where if they took it in the clinic, they can say, “Oh, yeah, I swallowed those yesterday, and it was fine. And so, therefore, now I’m at home. I can take that.” [HIV Primary Care_Provider_06]

#### Messaging to the patient

A key commonality in the messaging strategies participants discussed was their purpose in emotionally supporting and partnering with a Rapid ART patient. Some messages aimed to show support directly, and some used more subtle expressions of partnership (i.e., using the word “we” instead of “you”). A trust-building strategy mentioned by multiple participants was a clinician or member of the Rapid ART team disclosing their own HIV status and talking about their own experiences with HIV treatment. One participant described the impact of this messaging on a patient who had initially declined Rapid ART, but ultimately changed her mind:

I oftentimes share about my own diagnosis. I’ve been living for 24 years with it. It’s like the people know, uh, and it’s empowering to them, right. And so oftentimes I’m like the only other person living with HIV who they had a chance to talk to. You know, and, like that one person who [declined Rapid ART at first], when I saw her, I think it was like the week afterwards that I took care of her and, you know, we were talking about, and I disclosed to her. And, uh, she kind of broke down. She’s like, you’re the first person I’ve met who has like told me that. I was like, [there are] tons of people out there who are living with HIV. [Testing Site_Provider_04]

Another provider gave examples of the “little things” that their team does and says to build trust and relationships with clients during the Rapid interaction:

We’ll be like, you know, okay, “Do you have a cellphone? Okay, here it is. Okay, what emoji do you want by your name?” You know, like, little things like that to make the whole process just more humanized. … ‘Cause it’s all about relationship building. Like, it’s one thing to prescribe meds, and it’s a completely different thing to help support our patients, like, swallow the meds. [HIV Primary Care_Provider_04]

##### Key take-aways for implementation

➣ Pair clinical support with psychosocial support and partnership. This is partly about providing comfort, but also the idea that the message of the Rapid interaction isn’t *you leave here and do this thing on your own*, but rather, *your treatment is a partnership with us and we can start that together, right here*.

➣ Find ways to humanize the interaction. It can be something big, like disclosing one’s own HIV status or experience with loved ones living with HIV, or it can be small, like offering personalized communications strategies.

➣ Don’t leave patients alone during the initial Rapid ART visit if at all possible.

### Correcting misperceptions about HIV

#### Conceptual approach

Much of the approach and messaging reported within the Rapid interaction was grounded in the awareness that clients have an existing understanding about HIV, and what an HIV diagnosis means. That understanding could be current and based on knowledge and experience with people living with HIV today, or it could be incorrect or outdated, reflective of the stigma and medical prognoses that were the hallmark of HIV in its earlier days. Key and common to the Rapid interactions reported by participants was correcting misperceptions of HIV, and the framing (or reframing) of HIV as a chronic condition like many others that needs management, but that need not limit a person’s life.

There’s such a variance of people who are testing positive. Some people anticipate it, others do not. It’s the last thing on their mind. Some people know a lot about HIV, have been in the community a long time, know a lot of positive people, know how it’s shifted and changed and what the reality is today. Some people coming here from other places or just even out of county, I mean, it’s astonishing to me just the further you - how little people know … and how vivid and intense how it was 30, 40 years ago still is, that is the image that remains. Doesn’t matter what has changed since then. And it’s seared into people’s subconscious, so it is so common when people hear you are HIV positive, it’s so common for it still to be, oh, my God, I’m going to die. [Testing Site_Staff_03]

This theme overlaps with patient-centeredness and emotional support, in that participants reported modulating their messaging based on what individual clients needed, as demonstrated in finding 1, and that correcting misperceptions of HIV was a tool of comfort and support.

It’s kind of [I] think one of the most intimate situations to have with a client. I kind of think it’s almost on the level of delivering a baby. Somebody just had something that has changed their life disclosed to them, um, and being able to be there to help support and give accurate information. And help them frame it in a way that, you know, their life is not going to be shortened or really, really altered by this. [Testing Site_Staff_07]

#### Messaging to the patient

There were some phrases that participants repeated almost verbatim across interviews—clusters of phrases that they used with patients to destigmatize what living with HIV means today and reassure patients in the face of the fears this new diagnosis can bring. These main messages came up again and again: you can live a normal life; you can have a healthy sex life; undetectable means untransmittable (U = U); you can still have biologically-related children; this isn’t a death sentence.

And then I usually say something to effect of, I would just like to name the elephant in the room. And I just want to speak these words out loud with you that HIV does not mean that you’re going to die. It does not mean that you can’t have a full and healthy sex life. It does not mean that you can’t have biological children if you want them. It does not mean any of those things anymore. All it means is that you have to take medicine every day, and see a doctor regularly. [FQHC_Provider_05]

I’ll usually say something to the effect of, it’s kind of similar to having diabetes or high blood pressure. As long as you manage it and you take care of yourself, it generally doesn’t affect your overall quality of life or longevity. [FQHC_Staff_01]

##### Key take-aways for implementation

➣ Many people’s fears and anxieties about HIV are grounded in outdated information.

➣ Normalize HIV as a controllable, treatable chronic condition like many others to inform and reassure the patient, and to help combat stigma.

➣ Provide education about accurate, current science related to HIV—treatment options, benefits of Rapid ART, risks associated with delayed versus immediate ART initiation, and reasonable lifestyle expectations—in an accessible format.

➣ Sample language of key messages: this is not a death sentence; you can have a healthy sex life; U = U; you can still have children; this is a manageable condition.

## Discussion

For as much as each provider honed their own approach to the Rapid interaction, it is striking that those seasoned in operationalizing Rapid ART reported approaches and messaging with such distinct commonalities. We found provider approaches to be anchored in themes of patient-centeredness, emotional support and partnership with patients, and framing HIV as a treatable and manageable condition with early treatment initiation.

It is important to note that these findings may not be *unique* to the Rapid interaction—we do not suppose that these approaches or strategies have never before been employed within a traditional timeline of care—but they are *specific* to it. Rapid ART is a process comprised of many of the same steps involved in traditional HIV care and ART initiation (e.g., diagnosis, counseling, education, benefits navigation, linkage, treatment selection, medication procurement, and initiation support), compressed, ideally, into a single visit. As these services are compressed, so too are the needs of the patient, creating a unique constellation of considerations and responsibilities for a provider. This visit is distinct from a clinical encounter involving an HIV diagnosis alone, or a post-diagnosis follow-up visit to discuss treatment options when a client has known their diagnosis for several days, weeks, or (in some cases of delayed appointment availability) months. Any echoes in our findings of themes common to conventional HIV diagnosis, linkage, and care strategies should be viewed as evidence that Rapid ART is an accessible practice, utilizing many of the same competencies already developed by those working in HIV testing and treatment.

It is a main limitation of this study that while we can present provider approaches and identify commonalities across participants and sites, we are unable to assess whether these approaches contributed to positive experiences for patients or towards improved clinical or behavioral outcomes. We did collect patient data under the broader study umbrella, but because patients and providers were not interviewed in dyads, we cannot infer conclusions about whether patients experienced staff’s and providers’ care approaches as they were intended. Furthermore, the patient behavioral outcome data that were collected are associated with the application of Rapid ART as a standard of care broadly, and cannot be attributed directly to any one component or level of implementation.

That said, our findings are not wholly without validation from the patient perspective. In this study, the iterative process of honing approaches to the Rapid interaction was driven in large part by what staff and providers perceived to be effective for their patients. Though not to be conflated with patient reports of their own experiences, provider perception of patient experience is a valid data construct within the CFIR; our interview guide specifically queried provider perception of patient needs and experience as a construct of the outer setting.

Further, patient data from this study were analyzed separately [39], and without inferring direct links between meaningful care elements that patients reported and those intended by providers, we can broadly identify concordance across findings. Patient findings published in 2021 show that, in addition to the offer of the ART itself, patients valued the emotional support of the “ART encounter,” and that Rapid ART as a process helped offset patient fears [39].

Finally, we see concordance between our findings and existing literature on what patients report as having been valuable elements of their experiences with HIV providers broadly, and Rapid ART specifically. Evidence suggests that paternalistic or provider-centric approaches are barriers to engagement in HIV care and that perceptions of a “care partnership” [40] and “provider warmth and affirmation” [30] are powerful facilitators to patient engagement and retention. Two 2013 qualitative studies exploring patient perspectives on new HIV diagnoses and engagement in care stress the importance of emotional support from providers, building trust, and messaging that highlights HIV as a manageable chronic condition [41, 42]. A 2020 qualitative study of patients’ experiences of access to same-day ART suggested high patient acceptability of the model and highlighted the patient-perceived value of emotional support from trusted providers and the power of “important conversations” with providers to assuage fears [43].

## Conclusion

Exploration of provider-level approaches to Rapid ART implementation, as carried out in the patient-provider Rapid interaction, contributes a critical layer of evidence for wider implementation. It is our hope that, together with existing research showing positive outcomes and core components of systems-level implementation, these findings add to an instructive body of findings that facilitates the implementation of Rapid ART as an enhanced model of HIV care.

## Supplementary Information

**Additional file 1.** Progression of Theme Generation. Flow chart illustrates the progression of how themes were generated in the qualitative analytical process, then how they were organized and consolidated, resulting in the three major themes reported in the results section of this manuscript.

**Additional file 2.** Standards for Reporting Qualitative Research (SRQR) Checklist. Completed SRQR checklist, demonstrating how each item on the checklist was considered and/or addressed in the manuscript. This checklist is a manuscript submission requirement for qualitative research for this journal.

**Additional file 3.** Long-form provider narratives. This additional file provides an important supplement to our research findings. Rather than rely exclusively on excerpted quotes from participants, we highlight selected long-form narratives, where providers report on the fullness of their implementation experiences in their own words. Themes from our findings are interwoven throughout the narratives.

## Data Availability

The datasets generated and analyzed during the current study are not publicly available due to their potential for participant identification, but portions may be made available by the corresponding author upon reasonable request. We are not able to share this complete dataset more broadly without compromising participant confidentiality. Even within large institutions, these Rapid ART teams are quite small and often have only one or just a few individuals in each role. Several elements of each interview—be them contextual, references to job duties or roles, speech patterns and idioms, or elements we may not be able to anticipate or identify—could reveal the identity of the informant with a likelihood that makes it our responsibility to avoid. Furthermore, making the full content of these interviews publicly available is beyond the scope of data use to which participants consented.

## Abbreviations

ART: Antiretroviral therapy
CFIR: Consolidated framework for implementation research
FQHC: Federally qualified health center
HIV: Human immunodeficiency virus
RAPID: Rapid ART Program for Individuals with an HIV Diagnosis
STI: Sexually transmitted infection
U = U: Undetectable = Untransmittable (e.g., a person with an undetectable HIV viral load cannot transmit the HIV virus to others)
