# Supplementary material for: The Rapid interaction: a qualitative study of provider approaches to implementing Rapid ART
Source: Implement Sci Commun. 2023 Jul 14;4:78. doi: 10.1186/s43058-023-00464-w (PMC10349523; doi:10.1186/s43058-023-00464-w)
Supplement: Supplementary file 1 — Additional file 1. Progression of Theme Generation. Flow chart illustrates the progression of how themes were generated in the qualitative analytical process, then how they were organized and consolidated, resulting in the three major themes reported in the results section of this manuscript. [file 43058_2023_464_MOESM1_ESM.docx]

Additional File 1. Progression of Theme Generation

*Provider interview code review*

- RP_Pro_how
- Comm
- PR_attitude
- PR_attitude_advan
- PR_accept
- Pt_accept
- Pt_needs
- Recd
- QQ

*Provider interview code excerpting*

- RP_Pro_how
- Comm
- PR_attitude
- PR_accept

*3 Themes*

- Patient-centeredness
- Emotional support and partnership
- Correcting misconceptions about HIV

*5 Themes*

- Patient-centeredness
- Handholding
- Trust- and partnership-building messages
- Destigmatizing and reassuring messages
- Education

*HIV Primary Care Site themes*

- Explaining the science
- Bridge from clinic to home
- ART as action / empowering
- Provider models importance / urgency
- Patient-centered
- Approaches to RAPID offer
- Humanizing
- Unexpected / radical statement
- Provider confidence / guidance
- Provider disclosure
- Gave a “good feeling”

*Testing Site themes*

- Client-centered / client-led
- Psychosocial support
- Hand-holding (logistical)
- “You can live a normal life”
- U=U
- Harm reduction / responsibility to clients
- Urgency

*FQHC themes*

- Psychosocial support
- Education
- Patient-centered / collaborative
- “This is not a death sentence”
- U=U
- Inspire confidence
- Scientific progress

*Second pass theme summarizing*

- Trust, rapport, and partnership (radical statements, “elephant in the room,” provider disclosure)
- Comfort (hope for the future, good feeling)
- Confident care
- RAPID as empowering action
- Essential messages (not a death sentence, children, normal life, U=U)
- Hand-holding (bridging clinic and home, emotional support)
- Explanation of the science

*First pass theme summarizing*

- Patient-centered (What does this client need? What are their questions? What is their head space?)
- Addressing “elephant in the room” (death sentence, stigma)
- Hope for the future
- Good feeling
- Provider confidence: “you’re in good hands”
- Urgency
- Actionable / empowering
- “You can live a normal life”
- “This is not a death sentence”
- “You can have children”
- “Undetectable means untransmissible”
- Handholding (bridging clinic and home)
- Psychosocial / emotional support
- Explanation of the science
- Provider disclosure
- Radical / surprising statements
