# Supplementary material for: The Rapid interaction: a qualitative study of provider approaches to implementing Rapid ART
Source: Implement Sci Commun. 2023 Jul 14;4:78. doi: 10.1186/s43058-023-00464-w (PMC10349523; doi:10.1186/s43058-023-00464-w)
Supplement: Supplementary file 2 — Additional file 2. Standards for Reporting Qualitative Research (SRQR) Checklist. Completed SRQR checklist, demonstrating how each item on the checklist was considered and/or addressed in the manuscript. This checklist is a manuscript submission requirement for qualitative research for this journal. [file 43058_2023_464_MOESM2_ESM.docx]

## Additional File 2. Standards for Reporting Qualitative Research (SRQR) checklist

<https://www.equator-network.org/reporting-guidelines/srqr/>

| Number | Topic | Item | Description / line number |
| --- | --- | --- | --- |
| **Title and Abstract** | | | |
| S1 | Title | Concise description of the nature and topic of the study identify the study as qualitative or indicating the approach or data collection methods. | The Rapid interaction: a qualitative study of provider approaches to implementing Rapid ART  [lines 1, 2] |
| S2 | Abstract | Summary of key elements of the study using the abstract format of the intended publication. | Lines 24-52 |
| **Introduction** | | | |
| S3 | Problem Formulation | Description and significance of the problem/phenomenon studied; review of relevant theory and empirical work; problem statement | Background section, lines 82-93 |
| S4 | Purpose or research question | Purpose of the study and specific objectives or questions. | In study aims, lines 124-131 |
| **Methods** | | | |
| S5 | Qualitative approach and research paradigm | Qualitative approach and guiding theory if appropriate; identifying the research paradigm is also recommended; rationale | The theoretical and practical rationales behind the selection of the Consolidated Framework for Implementation Research (CFIR) as a basis for the qualitative analysis is explained in detail.  See lines 133-166 |
| S6 | Researcher characteristics and reflexivity | Researchers’ characteristics that may influence the research, including personal attributes, qualifications/experience, relationship with participants, assumptions, and/or presuppositions; potential or actual interaction between researchers’ characteristics and the research questions, approach, methods, results, and/or transferability | Affiliations are listed on the title page (not in the blinded version), and (blinded) author contributions are listed at the end of the Declarations section at the end of the manuscript. Additional information about the roles and qualifications of the interviewers are included in the Methods section [lines 188-198, 222-226].  We did not discuss actual or potential interaction between researchers’ characteristics and research questions, approach, methods, results, and/or transferability. |
| S7 | Context | Setting/site and salient contextual factors; rationale | Methods section, lines 169-175 |
| S8 | Sampling strategy | How and why research participants, documents, or events were selected; criteria for deciding when no further sampling was necessary; rationale | Methods section, lines 181-186 |
| S9 | Ethical issues pertaining to human subjects | Documentation of approval by appropriate ethics review board and participant consent, or explanation for lack thereof; other confidentiality and data security issues | Declarations section, lines 533-538 |
| S10 | Data collection methods | Types of data collected; details of data collection procedures including start and stop dates of data collection and analysis, iterative process, triangulation of sources/methods, and modification of procedures in response to evolving study findings; rationale | Methods section, lines 181-186 |
| S11 | Data collection instruments and technologies | Description of instruments and devices used for data collection; if/how the instruments change over the course of the study | Methods section, lines 177-198  Interview guide available from author upon request. |
| S12 | Units of study | Number and relevant characteristics of participants, documents, or events included in the study; level of participation | Methods section, lines 200-210 |
| S13 | Data processing | Methods for processing data prior to and during analysis, including transcription, data entry, data management and security, verification of data integrity, data coding, and anonymization/deidentification of excerpts | Methods section, lines 212-239 |
| S14 | Data analysis | Process by which inferences, themes, etc. were identified and developed, including the researchers involved in data analysis; usually references a specific paradigm approach; rationale | Methods section, lines 212-239  Additional analytical process outlines in Additional File 1 |
| S15 | Techniques to enhance trustworthiness | Techniques to enhance trustworthiness and credibility of data analysis; rationale | Methods section, lines 228-235 |
| **Results/findings** | | | |
| S16 | Synthesis and interpretation | Main findings; might include development of a theory or model, or integration with prior research or theory | Results section, lines 241-452 |
| S17 | Links to empirical data | Evidence to substantiate analytic findings | Discussion section, lines 486-509 |
| **Discussion** | | | |
| S18 | Integration with prior work, implications, transferability, and contributions to the field | Short summary of main findings; explanation of how findings and conclusions connect to, support, elaborate on, or challenge conclusions of earlier scholarship; discussion of scope of application/generalizability; identification of unique contributions to scholarship in a discipline or field | Discussion section, lines 455-474 |
| S19 | Limitations | Trustworthiness and limitations of findings | Discussion section, lines 476-484 |
| **Other** | | | |
| S20 | Conflicts of interest | Potential sources of influence or perceived influence on study conduct and conclusions; how these were managed | Declarations section, line 556 |
| S21 | Funding | Sources of funding and other support; role of funders in data collection, interpretation, and reporting | Declarations section, line 559-563 |
