# Supplementary material for: The Rapid interaction: a qualitative study of provider approaches to implementing Rapid ART
Source: Implement Sci Commun. 2023 Jul 14;4:78. doi: 10.1186/s43058-023-00464-w (PMC10349523; doi:10.1186/s43058-023-00464-w)
Supplement: Supplementary file 3 — Additional file 3. Long-form provider narratives. This additional file provides an important supplement to our research findings. Rather than rely exclusively on excerpted quotes from participants, we highlight selected long-form narratives, where providers report on the fullness of their implementation experiences in their own words. Themes from our findings are interwoven throughout the narratives. [file 43058_2023_464_MOESM3_ESM.docx]

## Additional File 3. Long-form provider narratives

**Narrative 1: Not the death sentence it was 30 years ago**

I literally just give them the results. I will just look at them and say, "So, it does look like according to our tests, you tested positive for HIV."

And then I say, "I'm going to give you a moment to kind of think about that, and when you're ready to start talking about that, just let me know." Oftentimes, the patients will appreciate a moment to kind of let that sink in a little bit. Once they're ready to talk, I basically say that "HIV is a disease that has a very large stigma attached to it and a stigma that it doesn't really deserve anymore with where we're at in terms of treating it."

I talk them through how much progress we've made in treating [with] medication that while it isn't curable, it's completely controllable. That most folks who are in care are going to end up getting an undetectable level of the virus pretty quickly after starting the anti-retroviral therapy that we want to get them started on and that HIV at this point in 2019 almost 2020, is by no means the death sentence it was 30 years ago.

That we're going to walk them through every process every step of the way to make sure that they get to undetectable, that you know once you're undetectable it's not going to affect your life expectancy. It's not going to affect your overall health, and U equals U, untransmittable, undetectable equals untransmittable, excuse me.

Just really trying to get to the main stigma, things that people have associated with the disease to make sure that they feel very comfortable knowing that they're in good hands with people that really know the virus really well and that they don't need to be you know, that they can ask any questions that they might have. My main spiel is basically, just to confront a lot of the false ideas people may have around HIV now because it's very different than it once was.

Even like five years ago, we were treating it differently, so kind of providing them with a lot of knowledge. Because from my perspective, anxiety and fear around these things generally come from lack of understanding, and so just knowing that I have a good, firm understanding of it so that I can answer their questions and provide them with a firm understanding of where they're at.

FQHC_PR04

**Narrative 2: It's really just about making people feel safe, and seen, and comfortable**

Meet them at the elevator. Like, kind of roll out the red carpet, truly. I mean, I put myself through nursing school and college with, like, I was a hostess. I worked in a service industry, and 100% of those skills apply, you know? It's really just about making people feel safe, and seen, and comfortable.

So, they come and check in. You know, they bring them to the [program] office, and then it's either one of the social workers that's doing the intake. It's most of the time one of the social workers that's doing the intake unless both of them are busy, or they're doing another intake, and then it would be - and then I would do the intake. And so, you know, checking with them. See how they're doing. Like, there's a series of questions of, like, you know, housing, and substance use, and, you know, medical history, and mental health history. And, like, how do they think this happened? What were risk factors? What do they know? Were they on PrEP? Were they not on PrEP? Like, what's their food security situation like? Do they get any sort of SSI, SSDI? Do they have a job? But not in that kind of, like, checkbox way. It's, like, you sit down, and you have a conversation with them. Like, I always have a jar of tea in my desk and snacks in my desk, and it's like, "Are you hungry? Can I make you a cup of tea?” It's really just about making them feel comfortable, so get through that.

And then one of the providers will come in after that, or we'll bring the patient to the provider room, and then we'll typically, the three of us, sit down, and, like, that's when we'll go in more extensively around, like, patient education around, you know, what the medications do, and viral load, and CD-4, and all the time really kind of just, like, gauging how the patient is doing because this is a ton of information, and so it's, like, wanting to make sure they're not too overwhelmed, and that they're, like, at least kind of taking in some of the information. And then make sure they get labs, as well, talk about the starter pack, make sure they have all of the things that they need, and then talk about that it's going to be a bunch of appointments at first because we're also teaching them how to engage in the healthcare system maybe for the first time. And for a lot of, like, especially our younger patients that have been totally healthy up until now and don't know what it's like to come into a primary care clinic, you know, once a month for the first three months or, you know, more than that or, you know, until it's - until they're a little bit more established, and we know that their viral load is suppressed or getting there. Then we can, like, space that out a little bit. So, we kind of set the stage of, like, expectations around how long that, like, how many times we're going to see them and, like, why it's important.

And then one of the things that we did when I started is, I got us a smartphone. I got us an iPhone, and so we, like, texting is a huge thing now because everybody texts. You know, it's little things, too. Like, we'll be like, you know, okay, put in there, "Do you have a cellphone? Okay, here it is. Okay, what emoji do you want by your name?" You know, like, little things like that to make the whole process just more humanized. And then talk about how, you know, we'll talk about how we'll follow up with them, you know, in the next day, next couple days, to make sure that they pick up their medication and just really kind of start to establish just the relationship with them 'cause it's all about relationship building. Like, it's one thing to prescribe meds, and it's a completely different thing to help support our patients, like, swallow the meds. Yeah.

HIV Primary Care_PR04

**Narrative 3: What’s your biggest worry right now?**

You know, so my approach to it originally is, I come into the room and I just say, your test result came back positive, and then I wait and see. And there’s a moment of quiet, and then there’s going to be two different ways. People are going to break down and cry a lot, or some people are like, I want to get down to business. Okay. What do I got to do next? And for me it’s just kind of keeping it client-centered and, um, kind of addressing their needs.

‘Cause my very next question is like, what’s the biggest thing on your mind, or what’s your biggest worry right now? ‘Cause I have my worries as a provider, but they’re not necessarily going to be their worries. You know, so I want to find out. It’s like is it ‘cause of your living situation? Who do you have to tell? Like how - you know, we have people who are applying for Green Cards and stuff like that, and they worry that HIV impacts that. Thankfully it doesn’t anymore. Um, so I kind of work with them and kind of deal with their priorities of what they have.

And I always make sure that by the end of that counseling session that they know that they have access to care, that they know their medications … that their life expectancy will be very - pretty much similar to the people who are HIV-negative. And then U equals U, which I think is very important.

So I make sure I kind of cover those essentials no matter what, in addition. And saying that someone could have like kids the old-fashioned way, like it’s empowering.

Testing Site_PR04

**Narrative 4: How do you get to undetectable?**

So it can be a fair amount of folks going in by the time I get there. But usually the conversation for me just sort of starts with a check-in. Like how are you doing? I know that this has been a busy day. You've seen a lot of people. How are you feeling? And just sort of start there and let them kind of lead the conversation. And then I usually do ask if they know folks living with HIV. Because again, I feel like that does pretty well anticipate their reaction toward the virus. If they say, oh, yeah, I've got five friends who are in grad school, becoming a lawyer and living with HIV is just an incidental part of their medical history.

Or like, no, I'm from Montana, I know no one living with HIV. All I know is the movie "Philadelphia," which is actually something a patient said to me once. The only thing I know about HIV is the movie "Philadelphia." Which means I'm going to have to do a lot more education on the front end. So I usually start there, kind of with like a knowledge track.

And then I usually say something to effect of, I would just like to name the elephant in the room. And I just want to speak these words out loud with you that HIV does not mean that you're going to die. It does not mean that you can't have a full and healthy sex life. It does not mean that you can't have biological children if you want them. It does not mean any of those things anymore. All it means is that you have to take medicine every day, and see a doctor regularly.

I know that some of these things may have been true in the past, but they are not anymore. And so I usually kind of like start with just clearing the air by naming that. And I think that kind of helps re-center people on what we're actually talking about. And then I'll usually say something to the effect of, it's kind of similar to having diabetes or high blood pressure. As long as you manage it and you take care of yourself, it generally doesn't affect your overall quality of life or longevity.

But I personally would rather be diagnosed with HIV than diabetes, because the lifestyle modifications with diabetes are intense. But I usually start there. And then I usually go into a brief explanation of viral load versus CD4. High versus low, what is high, what is low. How we get you low. How we get you undetectable. All of that stuff.

I talk briefly about U equals U. In talking about getting viral load to undetectable, what does that mean. Why do we care. And then that leads to like, how do you get to undetectable? You get to undetectable by taking your meds every day. I usually talk briefly about not rationing meds, not splitting them in half, things like that. It's a patient population that we care for does tend to be the type of person that is going to eventually have a point in their life where they're not going to have access to meds.

So I kind of like to talk to that on the front end. Like it's safer to stop meds entirely and start them again when you're in a financial space, head space, physical space where you can do that. Never split your meds. Never take one every other day to try and make them last. All of that kind of stuff. And then I kind of start to talk about the actual meds themselves.

We usually initiate most folks on Biktarvy. I don't really go into any detail about the method of action of the drug or anything like that. But I do kind of warn about some of the GI side effects that are possible. Talk about timing of taking it every day at the same time. And then I let them know that there's a slim chance the med might change at their follow-up appointment if we see something on labs that would indicate that that's needed.

I usually don't go much into detail on the med itself. Because I feel like, honestly, at that point, it's not that important. I've never had a patient name that that was important to them. They want something easy. They want something that's not going to make them feel like shit.

FQHC_PR05
